# Noninvasive assessment of renal function and fibrosis in CKD patients using histogram analysis based on diffusion kurtosis imaging

**DOI:** 10.1007/s11604-022-01346-2

**Published:** 2022-10-18

**Authors:** Guanjie Yuan, Weinuo Qu, Shichao Li, Ping Liang, Kangwen He, Anqin Li, Jiali Li, Daoyu Hu, Chuou Xu, Zhen Li

**Affiliations:** Department of Radiology, Tongji Hospital, Tongji Medical College, Huazhong University of Science and Technology, 1095 Jiefang Avenue, Qiaokou District Wuhan 430030, Hubei, China

**Keywords:** Magnetic resonance imaging, Diffusion kurtosis imaging, Histogram analysis, Renal function, Renal fibrosis

## Abstract

**Purpose:**

To investigate the potential of histogram analysis based on diffusion kurtosis imaging (DKI) in evaluating renal function and fibrosis associated with chronic kidney disease (CKD).

**Materials and methods:**

Thirty-six CKD patients were enrolled, and DKI was performed in all patients before the renal biopsy. The histogram parameters of diffusivity (*D*) and kurtosis (*K*) were obtained using FireVoxel. The histogram parameters between the stable [estimated glomerular filtration rate (eGFR) ≥ 60 ml/min/1.73 m^2^] and impaired (eGFR < 60 ml/min/1.73 m^2^) eGFR group were compared. Besides, patients were classified into mild, moderate, and severe fibrosis group using a semi-quantitative standard. The correlations of histogram parameters with eGFR and fibrosis scores were investigated and the diagnostic performances of histogram parameters in assessing renal dysfunction and fibrosis were analyzed. The added value of combination of most significant parameter with 24 h urinary protein (24 h-UPRO) in evaluating fibrosis was also explored.

**Results:**

Seven *D* histogram parameters in cortex (mean, median, 10th, 25th, 75th, 90th percentiles and entropy), two *D* histogram parameters in medulla (75th, 90th percentiles), seven *K* histogram parameters in cortex (mean, min, median, 10th, 25th, 75th, 90th percentiles) and three *K* histogram parameters in medulla (mean, median, 25th percentile) were significantly different between the two groups. The *D*_mean_ of cortex was the most relevant parameter to eGFR (*r* = 0.648, *P* < 0.001) and had the largest area under the curve (AUC) for differentiating the stable from impaired eGFR group [AUC = 0.889; 95% confidence interval (CI) 0.728–0.970]. The *K*_90th_ of cortex presented the strongest correlation with fibrosis scores (*r* = 0.575, *P* < 0.001) and achieved the largest AUC for distinguishing the mild from moderate to severe fibrosis group (AUC = 0.849, 95% CI 0.706–0.993). Combining the *K*_90th_ in cortex with 24 h-UPRO gained statistically higher AUC value (AUC = 0.880, 95% CI 0.763–0.996).

**Conclusion:**

Histogram analysis based on DKI is practicable for the noninvasive assessment of renal function and fibrosis in CKD patients.

## Introduction

Chronic kidney disease (CKD) shows gradually increased morbidity and mortality recently, thus becoming a worldwide public health problem [1]. Progressive decline of renal function may reach an endpoint of end-stage renal failure, which will make the patients undertake a high risk of death. Furthermore, renal fibrosis has consistently been shown to be the best predictor of progression in CKD [2]. Thus, timely and regularly monitoring of renal dysfunction and fibrosis is essential for guiding therapy and preventing the patients from poor prognosis [3, 4].

At present, the most common method for evaluating renal function is the estimated glomerular filtration rate (eGFR) based on serum creatinine (SCr) [5]. However, SCr is susceptible to various factors, which causes the restriction in the sensitivity of assessing renal function [6]. Renal biopsy is currently the gold standard of identifying the presence and extent of fibrosis, but it’s invasive, with the risk of severe complications, and should not be used for regular follow-up [7]. Hence, noninvasive and accurate techniques for the assessment of renal function and pathological progression are needed.

Diffusion-weighted imaging (DWI) is a noninvasive magnetic resonance imaging (MRI) technology that provides information about the movement of water molecules and is quantified by the apparent diffusion coefficient (ADC) [8]. Prior studies have demonstrated the potential of DWI to monitor renal function and pathological alteration noninvasively [9–11]. Conventional DWI follows a simple mono-exponential pattern based on Gaussian diffusion behavior without restriction [12]. However, water diffusion in living tissues is more complicated and is always restricted due to the presence of microstructures, such as cell membranes and organelles and extracellular matrix (ECM) in the fibrotic kidney tissue, namely non-Gaussian phenomena [13]. Thus, non-Gaussian model diffusion kurtosis imaging (DKI) was developed to provide greater sensitivity in tissue with microstructural complexity [13, 14]. This model evaluates the kurtosis (*K*) coefficient, which shows the deviation of tissue diffusion from a Gaussian approach, and the diffusivity (*D*) coefficient with the correction of non-Gaussian bias [15]. DKI may provide additional information and has shown promising performance in evaluating the alterations of renal function and assessing the degree of renal pathological injury of CKD in previous researches [16–18].

However, routine DKI signal measurements only provide mean values, which do not account for the underlying spatial distribution. Histogram analysis refers to the application of mathematical methods to analyze the relationship and distribution of pixel or voxel gray levels in the image, which reflects histologic characteristics and heterogeneity [19, 20]. Several studies have suggested that the histogram analysis of DKI is feasible for diagnosing and grading tumors and staging hepatic fibrosis [21–23], but until now the capability of DKI histogram analysis in reflecting the degree of renal dysfunction and fibrosis is not yet to be explored.

Therefore, this study aims to investigate whether histogram analysis based on DKI can be used to assess renal function and fibrosis associated with CKD.

## Materials and methods

### Patients

**Fig. 1:**
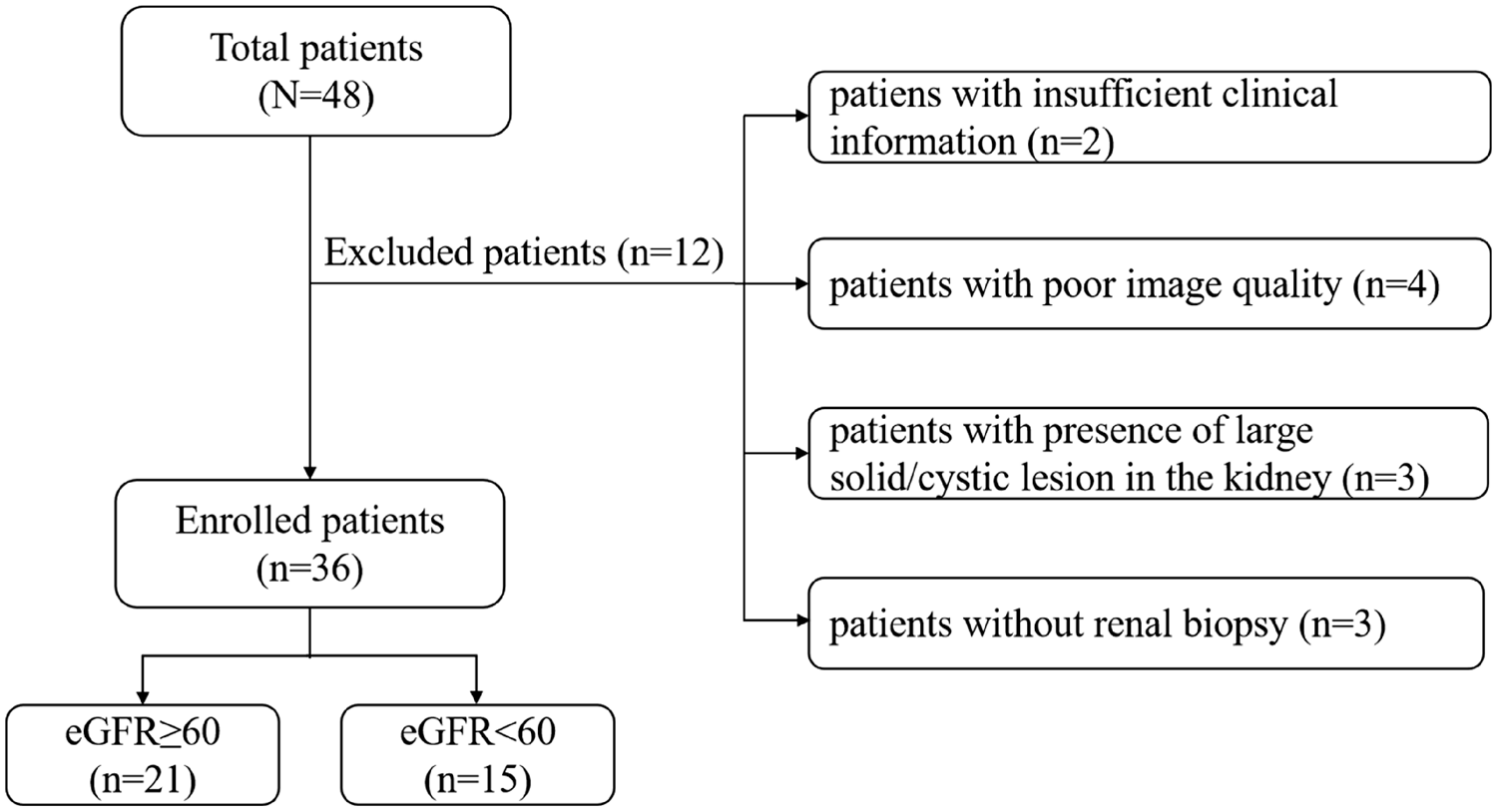
Flowchart of the study population

The Institutional Review Board of local hospital approved this study and the requirement for patient informed consent was waived. The CKD was defined based on 3 or more months of either kidney damage (blood or urine composition abnormalities, kidney biopsy findings, radiographic abnormalities) or eGFR lower than 60 mL/min/1.73 m^2^ [24]. This study retrospectively evaluated 48 adult patients who were diagnosed with CKD in the Department of Nephrology from August 2021 to January 2022. Among these participants, 12 patients were excluded for the following reasons: (a) insufficient clinical information (*n* = 2); (b) poor image quality (*n* = 4); (c) presence of large solid/cystic lesion in the kidney (*n* = 3); (d) without renal biopsy (*n* = 3). Finally, 36 CKD patients (16 males and 20 females; mean age: 41.5 ± 11.9 years; age range: 22–63 years) were successfully recruited for participation in our study. According to their eGFR value, patients were divided into two groups: group 1, patients with stable renal function (eGFR ≥ 60 ml/min/1.73 m^2^); group 2, patients with impaired renal function (eGFR < 60 ml/min/1.73 m^2^). The flowchart of study population is shown in Fig. 1.

### MRI acquisition

All MRI images were acquired using 3.0 T MR scanner (MAGNETOM Skyra, Siemens Healthcare, Erlangen, Germany) equipped with an eighteen-channel phased-array coil. All MRI examinations were performed within 1–2 days before the renal biopsy and the participants were required to fast for at least 4 h prior to the MRI examinations.

The conventional MR examination protocol were carried out, including: (a) coronal T2-weighted imaging (T2WI): repetition time [TR]/echo time [TE], 2240/67 ms; field of view [FOV], 360 mm*360 mm; matrix size, 320*256; slice thickness, 4.0 mm; (b) axial respiratory-triggered T1-weighted imaging (T1WI): TR/TE, 5.78/2.46 and 3.69 ms; FOV, 380 mm*380 mm; matrix size, 320*195; slice thickness, 3.0 mm; (c) axial T2WI: TR/TE, 3500/77 ms; FOV, 380 mm*380 mm; matrix size, 320*224; slice thickness, 4.0 mm.

DKI images were acquired using a free-breathing single-shot echo planar (SS-EPI) diffusion sequence in the axial plane. Five b-values (0, 500, 1000, 1500, and 2000s/mm^2^) in 4-directional diffusion-weighting gradient were used and the number of averages for each b-value was 1, 1, 2, 3 and 6, respectively. The other imaging parameters included: TR = 7700 ms, TE = 72 ms, slice thickness = 5.0 mm, FOV = 288*125 mm, Matrix = 120*120, bandwidth = 1666 Hz. The total acquisition time was 13 min.

### Image analysis

All the original images were transferred from the workstation into a personal computer and analyzed by an open-source software FireVoxel (https://fifiles.nyu.edu/hr18/public/ projects.html).

**Fig. 2:**
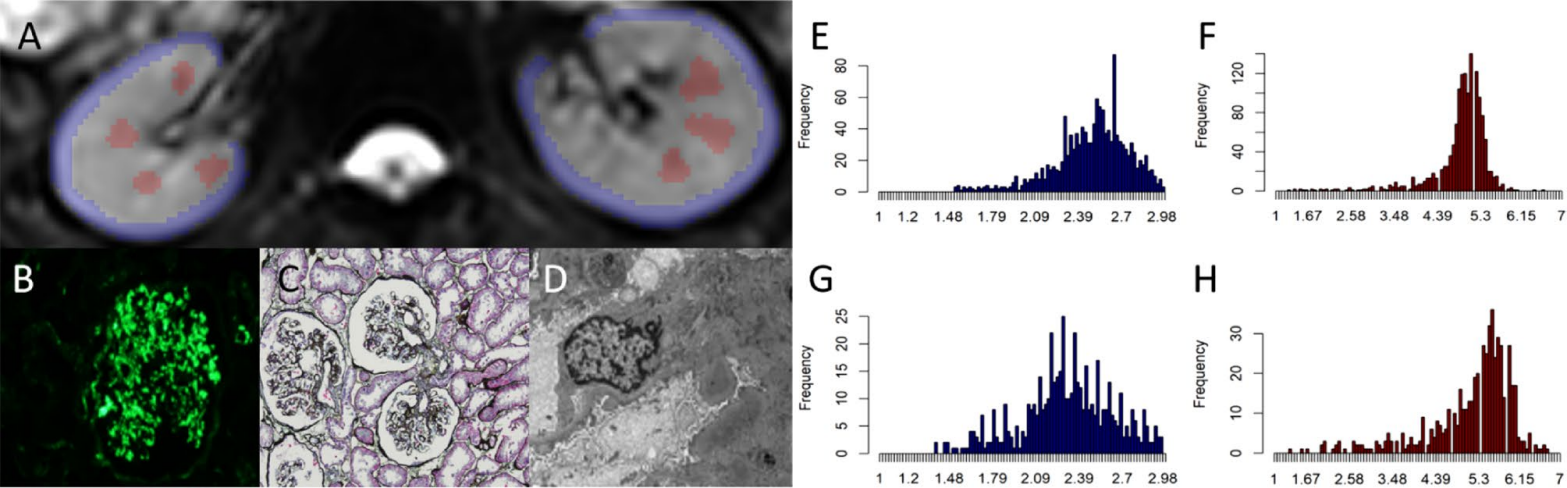
Female, 38 years, membranous nephropathy, estimated glomerular filtration rate (eGFR) = 117.5 ml/min/1.73 m^2^, interstitial fibrosis < 10%. **A** The regions of interest (ROIs) in cortex (blue) and medulla (red) on b = 0 s/mm^2^ image. **B**–**D**. The images of immunofluorescence, light microscopy, and electron microscopy, respectively. **E**–**H**. The gray-level histogram of diffusivity (*D*) in cortex, histogram of kurtosis (*K*) in cortex, histogram of *D* in medulla, histogram of *K* in medulla, respectively

**Fig. 3:**
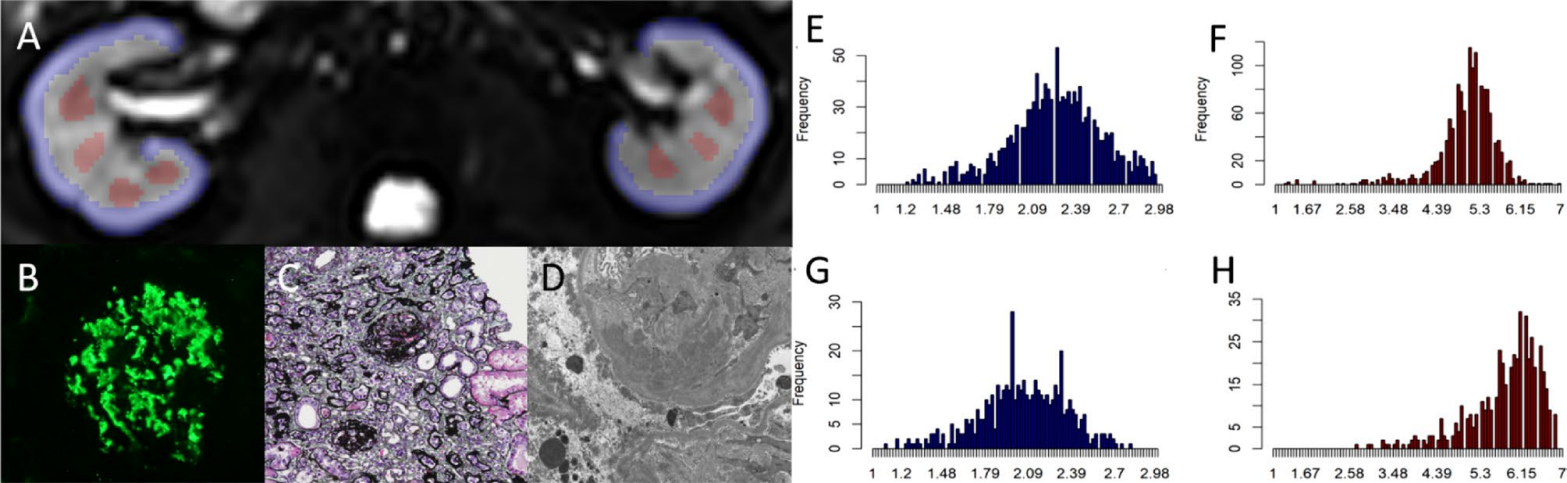
Male, 33 years, IgA nephropathy, estimated glomerular filtration rate (eGFR) = 40.8 ml/min/1.73 m^2^, interstitial fibrosis > 70%. **A** The regions of interest (ROIs) in cortex (blue) and medulla (red) on b = 0 s/mm^2^ image. **B**–**D**. The images of immunofluorescence, light microscopy, and electron microscopy, respectively. **E**–**H**. The gray-level histogram of diffusivity (*D*) in cortex, histogram of kurtosis (*K*) in cortex, histogram of *D* in medulla, histogram of *K* in medulla, respectively

Two radiologists (6 and 14 years of imaging experience in abdominal MRI) reviewed all the MR images, respectively, and they were both blinded to the clinical information of patients in this study. Radiologists manually draw the region of interest (ROI) in the renal cortex and medulla of bilateral kidneys at the largest level through the renal hilum on the images of b = 0 s/mm^2^. Taking the T2WI anatomical images as a reference, the cortical ROI was delineated around the outline of the kidney, and 4–8 ROIs were positioned on the medulla at the same (Figs. 2 and 3). All ROIs were placed to avoid vessels, renal sinus, renal cysts, and susceptibility artifacts carefully.

For the DKI model, diffusion-weighted signal intensities at multiple b values were based on the following formula: S(b) = S0∙exp(− b∙*D* + b^2^∙*D*^2^∙*K*/6), where S(b) is the signal intensity with diffusion weighting b, S0 is the signal intensity for a b value of 0 s/mm^2^, *D* represents the ADC after correction and *K* represents the degree to which the dispersion deviates from the Gaussian distribution [25]. For the delineated ROI of cortex and medulla, the software automatically extracted and calculated first-order parameters of *D* and *K* histograms, including mean, min, median, 10th, 25th, 75th, 90th percentiles, skewness, kurtosis and entropy.

### Demographic and clinicopathological parameters

Clinical information of all patients, including baseline characteristics, laboratory variables and pathological score were extracted from electronic medical records. All the patients underwent blood samplings within the week before the MRI examination to obtain the laboratory variables for assessing renal function including SCr, uric acid, blood urea nitrogen (BUN) and 24 h urinary protein (24 h-UPRO) value. The eGFR value was calculated using SCr based on the Chronic Kidney Disease Epidemiology Collaboration (CKD-EPI) [26]. All CKD patients received ultrasound-guided renal biopsy within 3 days after completion of the MRI examination. The pathological type and the extent of interstitial fibrosis were evaluated by a nephrologist with > 15 years of clinical experience in our hospital. A semi-quantitative method was used to assess the interstitial fibrosis (two-point scale): zero point was defined as < 25% mild fibrosis, one point was defined as 25–50% moderate fibrosis, and two points were defined as > 50% severe fibrosis.

### Statistical analysis

All statistical analyses were performed with SPSS version 25 statistical software (Chicago, IL, USA) and MedCalc (https://www.medcalc.org/). All tests were two-sided and values of *P* < 0.05 were considered statistically significant.

The intraclass correlation coefficient (ICC) with 95% confidence interval (CI) was used to assess the measurement consistency between two radiologists. Normality was assessed using the Shapiro–Wilk test (*P* ≥ 0.05 indicates normal distribution). Continuous variables were presented as mean ± standard deviations. The parameters between the stable and impaired eGFR group were compared by an independent sample Student *t*-test or Mann–Whitney *U* test and the histogram parameters between cortex and medulla were compared by paired *t*-test. Categorical variables were presented as number (percentage) and compared by the chi-squared analysis. Correlations of histogram parameters with eGFR and fibrosis scores were evaluated using Pearson correlation coefficient or Spearman rank correlation coefficient. Receiver-operating characteristic (ROC) curve and corresponding area under the curve (AUC) were used to determine the diagnostic performances of the histogram parameters in differentiating the stable from impaired eGFR group. Besides, the capabilities of histogram parameters in distinguishing the mild from moderate to severe fibrosis group were analyzed and the added value of combination of most significant parameter with clinical indicator 24 h-UPRO was also explored using ROC analysis.

## Results

### Patient characteristics

Out of all 48 CKD cases collected within the given time frame, a total of 36 patients who underwent MRI acquisition and renal biopsy successfully were enrolled in this study. The clinical and pathological characteristics of patients in the two groups are summarized in Table 1. All patients developed primary kidney disease and the most common pathological type was IgA nephropathy in the two groups. The patients in the impaired eGFR group were older than another group (*P* = 0.022). The Scr, uric acid, BUN and 24 h-UPRO in the impaired eGFR group were significantly higher than those in stable eGFR group (*P* < 0.001; *P* = 0.003; *P* < 0.001; *P* = 0.011, respectively), and the extent of interstitial fibrosis was significantly severer in the impaired eGFR group than in stable eGFR group (*P* = 0.005).

**Table 1:** The demographic and clinicopathological characteristics of patients in the stable and impaired eGFR group

Characteristics	eGFR ≥ 60 (n = 21)	eGFR < 60 (*n* = 15)	*P*
Male/female	10 (47.6%)/11(52.4%)	6 (40.0%)/9 (60.0%)	0.650
Age (years)	37.71 ± 10.64	46.80 ± 11.94	**0.022**
eGFR (mL/min/1.73 m^2^)	94.9 ± 22.6	36.6 ± 12.7	** < 0.001**
Serum creatinine (µmol/L)	80.00 ± 22.50	176.27 ± 44.26	** < 0.001**
Uric acid (mg/dL)	335.05 ± 83.59	432.45 ± 99.04	**0.003**
BUN (mg/dL)	5.62 ± 1.47	9.35 ± 2.92	** < 0.001**
24 h-UPRO (g/24 h)	1.19 ± 1.60	2.22 ± 1.66	**0.011**
*Pathological type of CKD*			0.413
IgA nephropathy	12 (57.1%)	12 (80.0%)	
Focal segmental glomerulosclerosis	3 (14.3%)	1 (6.7%)	
Membranous nephropathy	4 (19.0%)	2 (13.3%)	
Minimal change nephropathy	1 (4.8%)	0 (0.0%)	
Mesangial proliferative glomerulonephritis	1 (4.8%)	0 (0.0%)	
*Interstitial fibrosis*			**0.005**
< 25%	18 (85.7%)	5 (33.3%)	
25–50%	2 (9.5%)	5 (33.3%)	
> 50%	1 (4.8%)	5 (33.3%)	

Abbreviations: *eGFR* estimated glomerular filtration rate, *BUN* blood urea nitrogen, *24 h-UPRO* 24 h urinary protein, *CKD* chronic kidney disease

The bold indicated the items with statistically significant difference between the two groups

### Interobserver agreement

The degree of interobserver agreement was excellent (ICC > 0.81) for all histogram parameters, reflecting good repeatability. The ICC values for the histogram parameters of the cortex and medulla in *D* and *K* are presented in Table 2.

**Table 2:** The interobserver agreement between two radiologists for histogram parameters of *D* and *K* in the cortex and medulla

Parameters	Intraclass correlation coefficient		95% confidence interval	
	Cortex	Medulla	Cortex	Medulla
*D*				
Mean	0.949	0.932	0.877–0.977	0.858–0.967
Min	0.880	0.861	0.766–0.944	0.726–0.931
Median	0.937	0.913	0.873–0.969	0.811–0.959
10th percentile	0.924	0.945	0.840–0.964	0.847–0.977
25th percentile	0.934	0.932	0.858–0.968	0.862–0.967
75th percentile	0.967	0.937	0.932–0.984	0.864–0.970
90th percentile	0.951	0.917	0.902–0.976	0.836–0.959
Skewness	0.863	0.846	0.749–0.928	0.720–0.918
Kurtosis	0.842	0.863	0.711–0.916	0.750–0.928
Entropy	0.870	0.848	0.749–0.935	0.717–0.924
*K*				
Mean	0.962	0.944	0.923–0.982	0.888–0.972
Min	0.852	0.892	0.714–0.926	0.788–0.946
Median	0.980	0.949	0.958–0.990	0.897–0.975
10th percentile	0.946	0.952	0.892–0.974	0.885–0.979
25th percentile	0.969	0.938	0.937–0.985	0.877–0.970
75th percentile	0.976	0.967	0.951–0.989	0.933–0.984
90th percentile	0.981	0.964	0.960–0.991	0.926–0.982
Skewness	0.889	0.869	0.791–0.942	0.759–0.931
Kurtosis	0.877	0.875	0.774–0.935	0.761–0.936
Entropy	0.854	0.868	0.719–0.927	0.731–0.939

Abbreviations: *D* diffusivity, *K* kurtosis

### Comparisons of histogram parameters between cortex and medulla as well as between the different groups

**Fig. 4:**
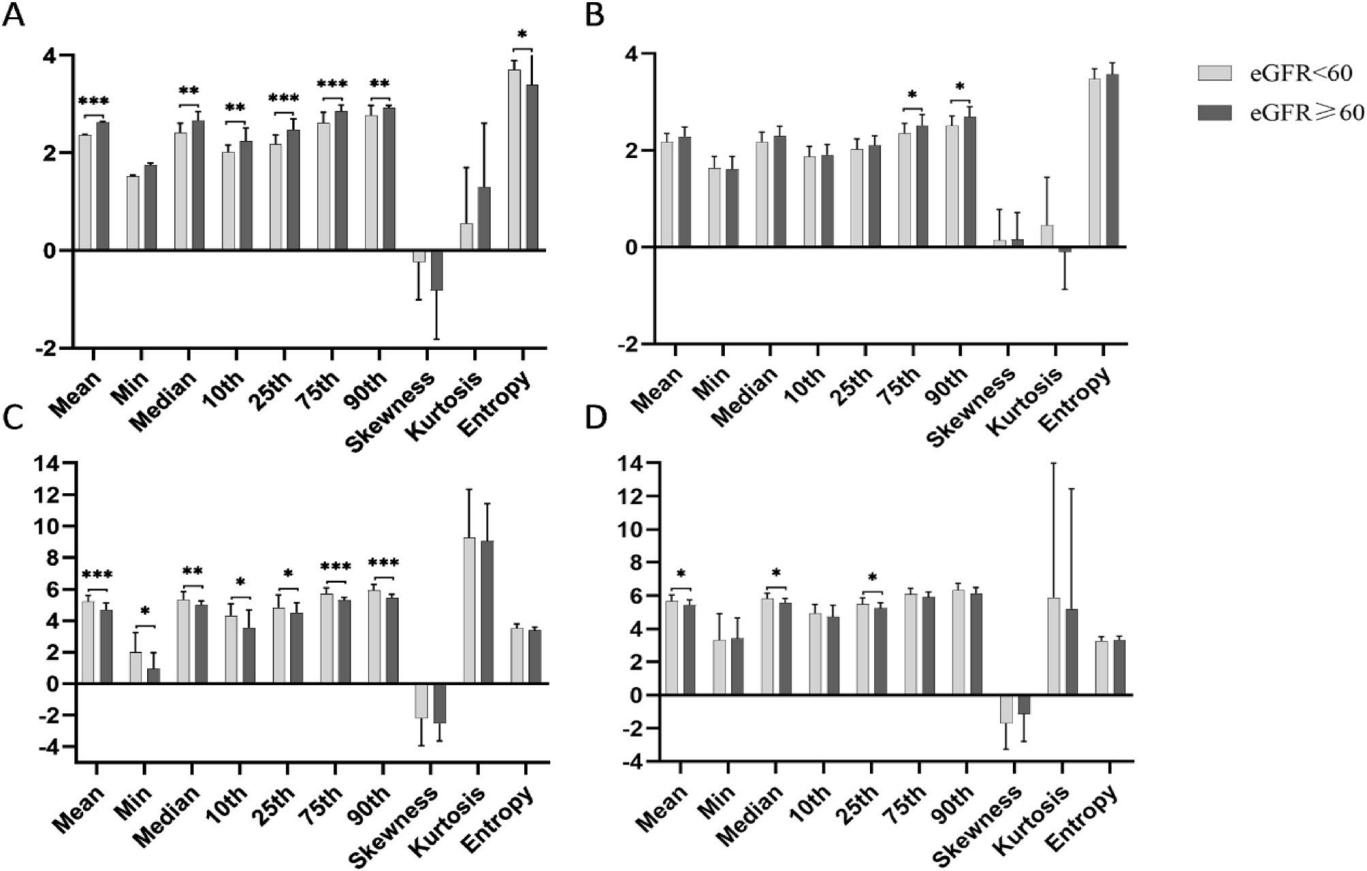
The comparisons of histogram parameters in cortex and medulla between the stable and impaired estimated glomerular filtration rate (eGFR) group. Bar charts comparing the histogram parameters of diffusivity in renal cortex (A) and medulla (B), the histogram parameters of kurtosis in renal cortex (C) and medulla (D) between the stable and impaired eGFR groups. *P < 0.05, **P < 0.01, ***P < 0.001

The results of comparisons between cortex and medulla as well as between the different groups are presented in Table 3 and Fig. 4. The values of mean, min, median, 10th, 25th, 75th, and 90th percentiles for *K* were significantly lower in cortex than medulla (all *P* < 0.001). Above mentioned histogram parameters except for min of *D* were significantly higher in cortex than medulla (all *P* < 0.001). In addition, the absolute values of skewness and kurtosis for *D*, the absolute value of skewness for *K* and the value of entropy for *K* were significantly higher in cortex than medulla (*D*: *P* < 0.001, *P* = 0.029; *K*: *P* = 0.012, *P* = 0.029, respectively).

**Table 3:** Comparisons of the histogram parameters between cortex and medulla as well as between the stable and impaired eGFR group

Parameters	Cortex			Medulla			*P* value
	eGFR < 60	eGFR ≥ 60	*P* value	eGFR < 60	eGFR ≥ 60	*P* value	
*D*							
Mean	2.366 ± 0.015	2.629 ± 0.017	** < 0.001**	2.172 ± 0.182	2.288 ± 0.194	0.087	** < 0.001**
Min	1.524 ± 0.019	1.753 ± 0.045	0.079	1.630 ± 0.246	1.615 ± 0.261	0.901	0.893
Median	2.419 ± 0.187	2.663 ± 0.188	**0.001**	2.176 ± 0.200	2.298 ± 0.204	0.117	** < 0.001**
10th percentile	2.012 ± 0.147	2.242 ± 0.270	**0.006**	1.871 ± 0.209	1.906 ± 0.214	0.634	** < 0.001**
25th percentile	2.180 ± 0.188	2.477 ± 0.218	** < 0.001**	2.024 ± 0.213	2.101 ± 0.200	0.486	** < 0.001**
75th percentile	2.613 ± 0.225	2.856 ± 0.128	** < 0.001**	2.358 ± 0.200	2.519 ± 0.224	**0.038**	** < 0.001**
90th percentile	2.765 ± 0.212	2.922 ± 0.057	**0.002**	2.511 ± 0.202	2.693 ± 0.211	**0.025**	** < 0.001**
Skewness	– 0.241 ± 0.767	– 0.816 ± 1.004	0.079	0.144 ± 0.640	0.160 ± 0.557	0.746	** < 0.001**
Kurtosis	0.555 ± 1.149	1.303 ± 3.197	0.929	0.452 ± 0.994	– 0.098 ± 0.768	0.083	**0.029**
Entropy	3.706 ± 0.188	3.390 ± 0.481	**0.011**	3.471 ± 0.219	3.578 ± 0.233	0.187	0.380
*K*							
Mean	5.235 ± 0.372	4.702 ± 0.427	** < 0.001**	5.685 ± 0.351	5.438 ± 0.301	**0.030**	** < 0.001**
Min	1.975 ± 1.285	0.934 ± 1.028	**0.011**	3.331 ± 1.593	3.415 ± 1.243	0.950	** < 0.001**
Median	5.333 ± 0.502	5.004 ± 0.247	**0.004**	5.819 ± 0.330	5.579 ± 0.247	**0.018**	** < 0.001**
10th percentile	4.313 ± 0.764	3.557 ± 1.118	**0.021**	4.932 ± 0.540	4.744 ± 0.689	0.800	** < 0.001**
25th percentile	4.821 ± 0.807	4.501 ± 0.640	**0.033**	5.490 ± 0.371	5.254 ± 0.324	**0.048**	** < 0.001**
75th percentile	5.705 ± 0.372	5.279 ± 0.202	** < 0.001**	6.089 ± 0.355	5.906 ± 0.312	0.110	** < 0.001**
90th percentile	5.920 ± 0.367	5.462 ± 0.222	** < 0.001**	6.335 ± 0.410	6.130 ± 0.357	0.120	** < 0.001**
Skewness	– 2.203 ± 1.748	– 2.539 ± 1.093	0.483	– 1.714 ± 1.538	– 1.139 ± 1.651	0.568	**0.012**
Kurtosis	9.284 ± 3.041	9.043 ± 2.386	0.950	5.894 ± 8.095	5.208 ± 7.245	0.950	0.100
Entropy	3.521 ± 0.278	3.387 ± 0.208	0.107	3.279 ± 0.247	3.349 ± 0.202	0.361	**0.029**

Abbreviations: *eGFR* estimated glomerular filtration rate, *D* diffusivity, *K* kurtosis

The bold indicated the items with statistically significant difference

For the histogram parameters of *D* in cortex, the impaired eGFR group have significantly lower mean, median, 10th, 25th, 75th, 90th percentiles and higher entropy than the stable eGFR group (*P* < 0.001; *P* = 0.001; *P* = 0.006; *P* < 0.001; *P* < 0.001; *P* = 0.002; *P* = 0.011, respectively).

For the histogram parameters of *K* in cortex, the impaired eGFR group have significantly higher mean, min, median, 10th, 25th, 75th, and 90th percentiles than the stable eGFR group (*P* < 0.001; *P* = 0.011; *P* = 0.004; *P* = 0.021; *P* = 0.033; *P* < 0.001; *P* < 0.001, respectively).

Besides, for the histogram parameters in medulla, significant differences were observed between the two groups in 75th, 90th percentiles of *D* and mean, median, 25th percentiles of *K* (medulla *D*: *P* = 0.038; *P* = 0.025; medulla *K*: *P* = 0.030, *P* = 0.018, *P* = 0.048, respectively). No significant differences were found in skewness and kurtosis of *D* or *K* in cortex and medulla between two groups (cortex *D*: *P* = 0.079, *P* = 0.929; medulla *D*: *P* = 0.746, *P* = 0.083; cortex *K*: *P* = 0.483, *P* = 0.950; medulla *K*: *P* = 0.568, *P* = 0.950, respectively).

### Correlations between histogram parameters and eGFR

**Fig. 5:**
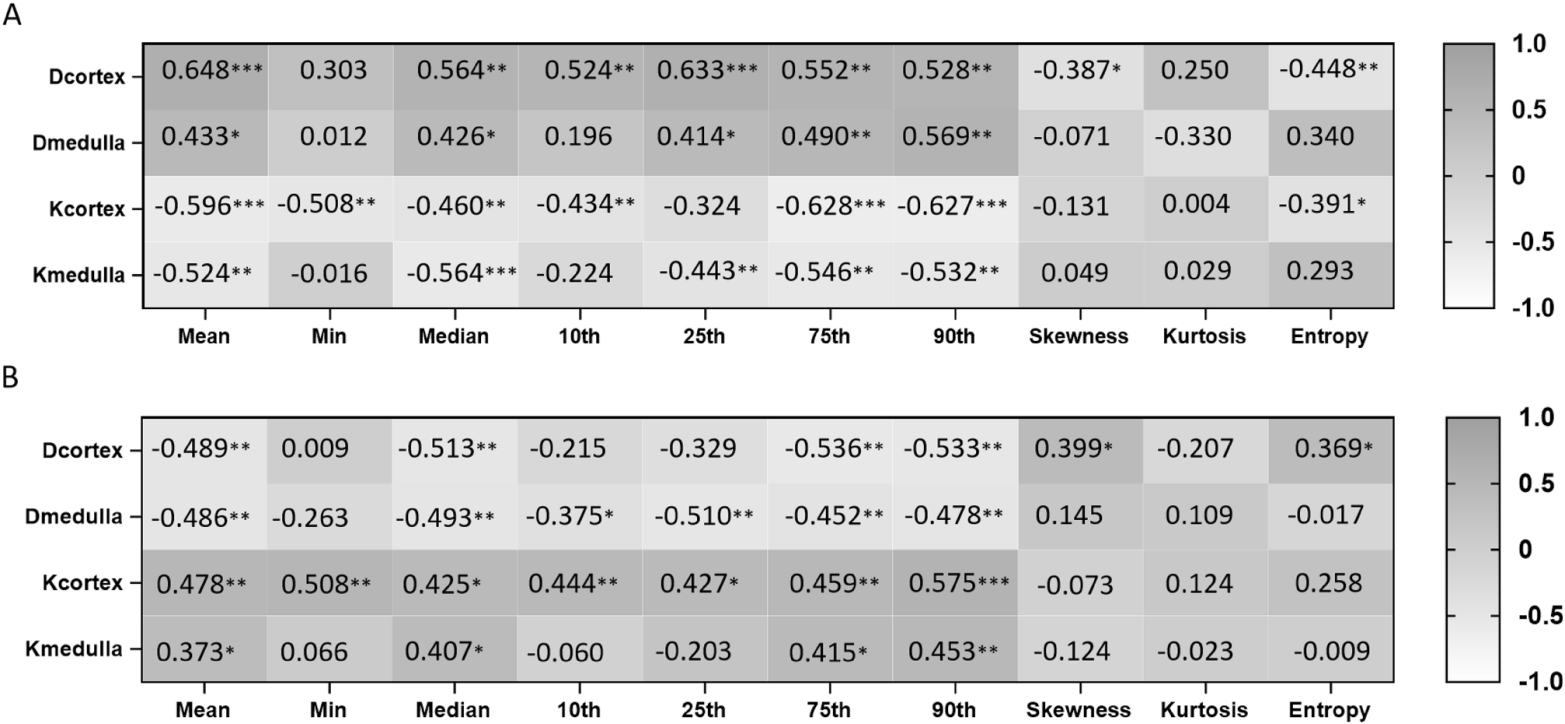
The relationships between the histogram parameters and the estimated glomerular filtration rate (eGFR) as well as between the histogram parameters and the histopathological fibrosis scores. These numbers represent correlation coefficients. A. The correlations between the histogram parameters and the eGFR. B. The correlations between the histogram parameters and the histopathological fibrosis scores. *P < 0.05, **P < 0.01, ***P < 0.001. Abbreviations: *D*, diffusivity; *K*, kurtosis

Among all histogram parameters, the mean value of *D* in cortex has the highest correlation coefficient with eGFR (*r* = 0.648, *P* < 0.001). Apart from skewness and entropy of *D* in cortex (*r* =  – 0.387, *P* = 0.026; *r* =  – 0.448, *P* = 0.009, respectively), the statistically significant histogram parameters of *D* in cortex and medulla were positively correlated with eGFR. Conversely, the statistically significant histogram parameters of *K* in cortex and medulla exhibited negative correlations with eGFR. The specific correlation coefficients and range of *P* values are exhibited in Fig. 5A.

### Correlations between histogram parameters and the histopathological fibrosis scores

Among all histogram parameters, the 90th percentile of *K* in cortex presents the strongest correlation with the histopathological fibrosis scores (*r* = 0.575, *P* < 0.001). Apart from skewness and entropy of *D* in cortex (*r* = 0.399, *P* = 0.021; *r* = 0.369, *P* = 0.018, respectively), the statistically significant histogram parameters of *D* in cortex and medulla were negatively correlated with the fibrosis scores. Conversely, the statistically significant histogram parameters of *K* in cortex and medulla were positively correlated with the fibrosis scores. The specific correlation coefficients and range of *P* values are exhibited in Fig. 5B.

### The performances of histogram parameters for assessing renal dysfunction and fibrosis

**Fig. 6:**
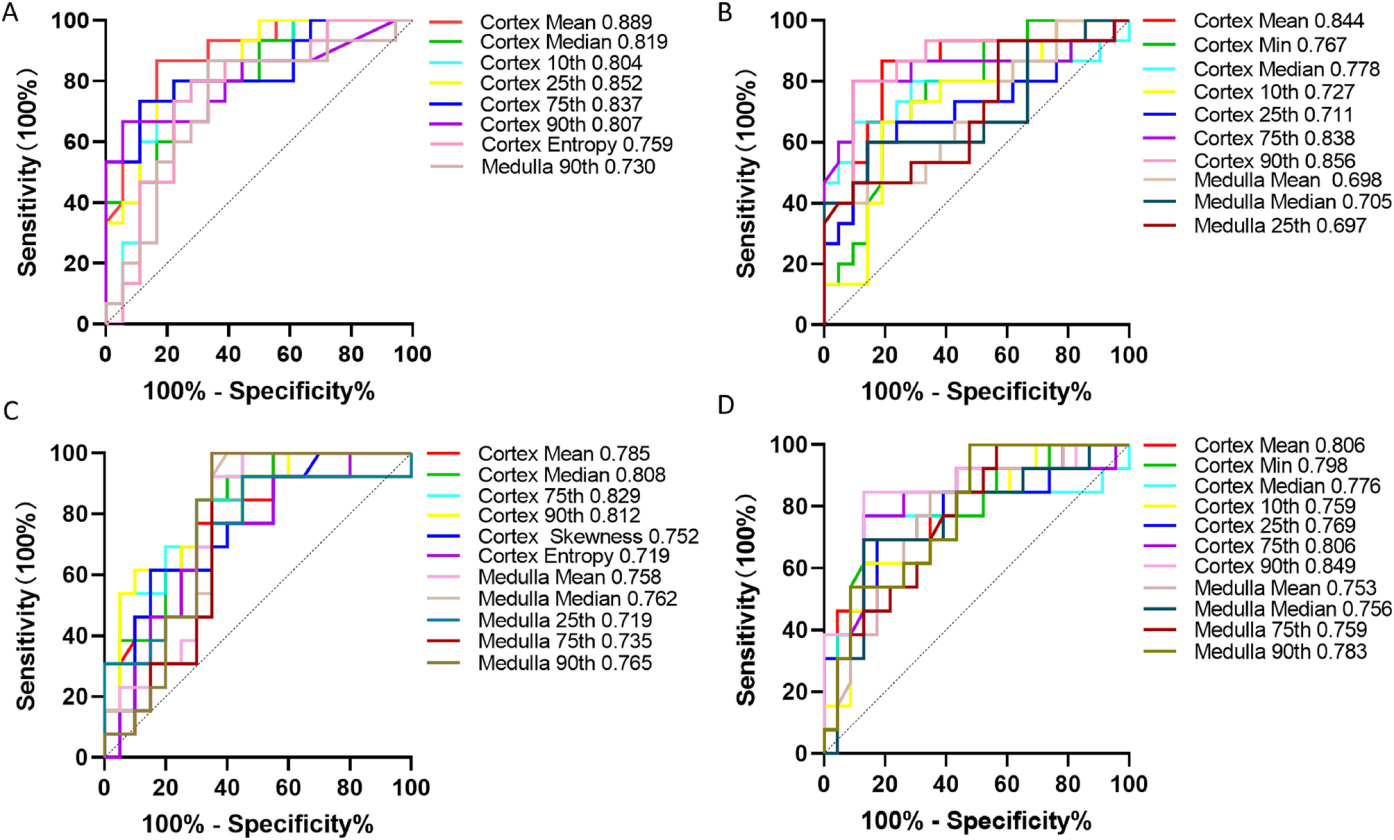
Receiver-operating characteristic (ROC) curves of the significant histogram parameters in discriminating different degrees of renal dysfunction and fibrosis. The ROC curves of the diffusivity (*D*) and kurtosis (*K*) histogram parameters in differentiating the stable from impaired estimated glomerular filtration rate (eGFR) group were shown in Fig. 6A and 6B, respectively. The ROC curves of the *D* and *K* histogram parameters in distinguishing the mild from moderate to severe fibrosis group were shown in Fig. 6C and 6D, respectively

**Fig. 7:**
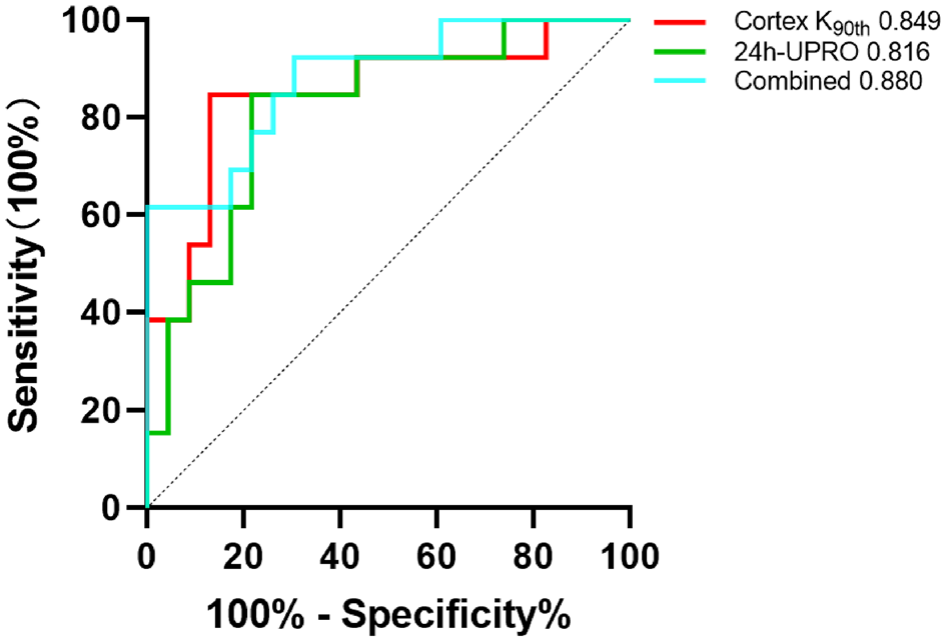
Receiver-operating characteristic (ROC) curves of the 90th percentile of kurtosis (*K*_90th_) in cortex, clinical indicator 24 h urinary protein (24 h-UPRO) and combination of them for distinguishing the mild from moderate to severe fibrosis group

As shown in Table 4 and Fig. 6, of the histogram parameters, the mean of *D* in cortex has the largest area under the curve (AUC) for differentiating the stable from impaired eGFR group [AUC = 0.889; 95% CI 0.728–0.970]. As shown in Table 5 and Fig. 6, of the histogram parameters, the 90th percentile of *K* in cortex has the largest AUC for distinguishing the mild from moderate to severe fibrosis group (AUC = 0.849, 95% CI 0.706–0.993). As presented in Table 6 and Fig. 7, Combining the *K*_90th_ in cortex with the value of 24 h-UPRO gained statistically higher AUC value for distinguishing the mild from moderate to severe fibrosis group than *K*_90th_ alone (AUC = 0.880, 95% CI 0.763–0.996) and corresponding sensitivity and specificity were 92.31% and 69.57%, respectively.

**Table 4:** Diagnostic performances of histogram parameters for differentiating the stable from impaired eGFR group

Parameters	Cortex				Medulla			
	AUC (95% CI)	Cutoff	Sensitivity (%)	Specificity (%)	AUC (95% CI)	Cutoff	Sensitivity (%)	Specificity (%)
*D*								
Mean	**0.889 (0.728–0.970)**	**2.477**	**86.67**	**83.33**	0.659(0.467–0.851)	2.271	80.00	61.11
Min	0.648 (0.456–0.840)	1.815	100.00	38.89	0.515 (0.311–0.719)	1.675	46.67	72.22
Median	**0.819 (0.676–0.961)**	**2.546**	**80.00**	**77.78**	0.663 (0.473–0.853)	2.281	80.00	61.11
10th percentile	**0.804 (0.629–0.921)**	**2.107**	**86.67**	**66.67**	0.541 (0.341–0.741)	2.092	93.33	22.22
25th percentile	**0.852 (0.724–0.980)**	**2.327**	**80.00**	**77.78**	0.574 (0.368–0.780)	2.013	66.67	61.11
75th percentile	**0.837 (0.694–0.980)**	**2.739**	**73.33**	**88.89**	0.700 (0.514–0.886)	2.451	80.00	66.67
90th percentile	**0.807 (0.648–0.967)**	**2.857**	**66.67**	**94.44**	**0.730 (0.549–0.910)**	**2.677**	**86.67**	**66.67**
Skewness	0.674 (0.546–0.857)	– 1.383	100.00	33.33	0.530 (0.349–0.705)	– 0.265	33.33	88.89
Kurtosis	0.511 (0.296–0.704)	– 0.129	20.00	55.56	0.659 (0.474–0.814)	0.103	60.00	72.22
Entropy	**0.759 (0.589–0.929)**	**3.565**	**80.00**	**72.22**	0.670 (0.475–0.865)	3.426	60.00	88.89
*K*								
Mean	**0.844 (0.711–0.978)**	**4.907**	**86.67**	**80.95**	**0.698 (0.521–0.876)**	**5.876**	**40.00**	**100.00**
Min	**0.767 (0.612–0.922)**	**2.015**	**80.00**	**66.67**	0.508 (0.337–0.678)	4.120	53.33	61.90
Median	**0.778 (0.600–0.956)**	**5.295**	**66.67**	**90.48**	**0.705 (0.521–0.889)**	**5.767**	**60.00**	**85.71**
10th percentile	**0.727 (0.557–0.897)**	**4.293**	**66.67**	**80.95**	0.527 (0.334–0.720)	4.270	86.67	28.57
25th percentile	**0.711 (0.527–0.895)**	**4.854**	**60.00**	**85.71**	**0.697 (0.516–0.878)**	**5.581**	**46.67**	**90.48**
75th percentile	**0.838 (0.681–0.995)**	**5.548**	**80.00**	**90.48**	0.638 (0.449–0.827)	6.273	40.00	95.24
90th percentile	**0.856 (0.663–0.988)**	**4.873**	**80.00**	**90.48**	0.651 (0.460–0.842)	6.445	46.67	90.48
Skewness	0.565 (0.363–0.767)	1.126	26.67	100.00	0.559 (0.354–0.763)	– 2.857	33.33	95.24
Kurtosis	0.508 (0.307–0.708	– 0.168	80.00	0.00	0.508 (0.301–0.715)	9.287	66.67	9.52
Entropy	0.654 (0.462–0.845)	3.528	53.33	80.95	0.552 (0.354–0.751)	3.244	46.67	76.19

Abbreviations: *eGFR* estimated glomerular filtration rate, *D* diffusivity, *K* kurtosis, *AUC* area under the curve, 95% CI 95% confidence interval

The bold indicated the items with statistically significant

**Table 5:** Diagnostic performances of histogram parameters for distinguishing the mild from moderate to severe fibrosis group

Parameters	Cortex				Medulla			
	AUC (95% CI)	Cutoff	Sensitivity (%)	Specificity (%)	AUC (95% CI)	Cutoff	Sensitivity (%)	Specificity (%)
*D*								
Mean	**0.785 (0.630–0.939)**	**2.521**	**84.62**	**65.00**	**0.758 (0.592–0.923)**	**2.271**	**92.31**	**65.00**
Min	0.538 (0.340–0.737)	1.379	92.31	40.00	0.627 (0.423–0.831)	1.353	38.46	95.00
Median	**0.808 (0.662–0.954)**	**2.548**	**84.62**	**70.00**	**0.762 (0.596–0.927)**	**2.338**	**100.00**	**60.00**
10th percentile	0.627 (0.435–0.819)	2.180	84.62	45.00	0.692 (0.506–0.879)	1.676	38.46	95.00
25th percentile	0.704 (0.525–0.883)	2.327	76.92	70.00	**0.719 (0.534–0.905)**	**2.166**	**92.31**	**55.00**
75th percentile	**0.829 (0.688–0.971)**	**2.873**	**100.00**	**55.00**	**0.735 (0.559–0.911)**	**2.474**	**100.00**	**65.00**
90th percentile	**0.812 (0.663–0.960)**	**2.835**	**61.54**	**90.00**	**0.765 (0.599–0.932)**	**2.712**	**100.00**	**65.00**
Skewness	**0.752 (0.630–0.962)**	**-0.090**	**61.54**	**85.00**	0.623 (0.438–0.785)	0.497	53.85	85.00
Kurtosis	0.644 (0.459–0.802)	0.016	61.54	70.00	0.585 (0.401–0.753)	– 0.311	69.23	55.00
Entropy	**0.719 (0.542–0.897)**	**3.565**	**76.92**	**65.00**	0.600 (0.385–0.815)	3.426	53.85	80.00
*K*								
Mean	**0.806 (0.656–0.956)**	**5.011**	**69.23**	**82.61**	**0.753 (0.590–0.915)**	**5.520**	**84.62**	**65.22**
Min	**0.798 (0.643–0.953)**	**2.036**	**69.23**	**82.61**	0.538 (0.332–0.745)	4.510	38.46	78.26
Median	**0.776 (0.585–0.966)**	**5.273**	**76.92**	**86.96**	**0.756 (0.583–0.928)**	**5.767**	**69.23**	**86.96**
10th percentile	**0.759 (0.597–0.921)**	**4.380**	**61.54**	**86.96**	0.508 (0.315–0.702)	5.028	69.23	47.83
25th percentile	**0.769 (0.602–0.936)**	**4.830**	**69.23**	**82.61**	0.647 (0.458–0.837)	5.574	46.15	82.61
75th percentile	**0.806 (0.642–0.970)**	**5.565**	**76.92**	**86.96**	**0.759 (0.604–0.915)**	**5.739**	**100.00**	**43.48**
90th percentile	**0.849 (0.706–0.993)**	**5.684**	**84.62**	**89.69**	**0.783 (0.633–0.933)**	**5.955**	**100.00**	**52.17**
Skewness	0.520 (0.302–0.738)	– 4.038	30.77	91.30	0.545 (0.342–0.749)	– 2.857	30.77	91.30
Kurtosis	0.554 (0.347–0.760)	17.314	30.77	91.30	0.532 (0.322–0.741)	2.102	61.54	65.22
Entropy	0.672 (0.479–0.865)	3.458	69.23	65.22	0.505 (0.314–0.696)	3.221	84.62	34.78

Abbreviations: *D* diffusivity, *K* kurtosis, *AUC* area under the curve, 95% CI 95% confidence interval

The bold indicated the items with statistically significant

**Table 6:** Diagnostic performances of K_90th_ in cortex, clinical indicator 24 h-UPRO and combination of them for distinguishing the mild from moderate-severe fibrosis group

Parameters	AUC (95% CI)	Cut-off	Sensitivity (%)	Specificity (%)	*P* value
Cortex *K*_90th_	0.849 (0.706–0.993)	5.684	84.62	89.69	0.002
24 h-UPRO (g/24 h)	0.816 (0.671–0.961)	1.476	84.62	78.26	0.002
Cortex *K*_90th_ + 24 h-UPRO	0.880 (0.763–0.996)	NS	92.31	69.57	< 0.001

Abbreviations: *K* kurtosis, *24* *h*-*UPRO* 24 h urinary protein, *AUC* area under the curve, 95% CI, 95% confidence interval

## Discussion

To our knowledge, this is the first study that performed the histogram analysis based on DKI to assess the renal dysfunction and fibrosis of CKD. Our study has demonstrated that several histogram parameters derived from *D* and *K* in cortex and medulla were significantly correlated with eGFR and fibrosis scores, and achieved good performance in discriminating different degrees of renal dysfunction and fibrosis.

Prior studies have confirmed that there are differences in *D* and *K* values between cortex and medulla, whether the subjects are normal people or patients with CKD [17, 18, 27]. In our study, the majority of histogram parameters of *D* were significantly higher in the cortex than medulla. This may be because the renal cortex has a higher blood supply and relatively more free water molecule diffusion than the medulla [28]. The more complex microstructure and the correspondingly greater deviation of water diffusion from the Gaussian form in medulla generate a higher *K* value [27], which is consistent with our results that most of the histogram parameters of *K* were significantly higher in the medulla than cortex.

Our study has found that the statistically significant histogram parameters of *D* (except for entropy in cortex) were lower in the impaired eGFR group than those in stable eGFR group, while the statistically significant histogram parameters of *K* were higher in the impaired eGFR group than those in another group, which was similar with the results of previous researches that the renal parenchymal mean *D* value would decrease and mean *K* value would increase as renal insufficiency progresses [17, 18, 29]. Furthermore, in our study, apart from skewness and entropy in cortex, the significantly positive correlations were observed between the great majority of *D* histogram parameters and eGFR, while most of the *K* histogram parameters tend to show significant negative correlations with eGFR. This may be attributed to the fact that the deterioration of renal function is accompanied by the progressive pathological alternation including glomerulosclerosis, tubular atrophy and interstitial fibrosis, which may lead to the decline of blood perfusion and more irregular microstructure [29]. Accordingly, the diffusion of water molecules was restricted and deviation from the Gaussian distribution became more serious. The glomerular filtration is susceptible to the blood perfusion, and the reduction of blood supply principally occurred in cortex rather than medulla considering around 90% of renal blood flow generally exists in cortical microvessels [30]. This may explain why the mean value of *D* in cortex have achieved the strongest correlation with eGFR in our study, which was consistent with the result that shown in the study of Mao et al. [17]. In addition, the mean value of *D* in cortex also exhibited the best performance in distinguishing two groups with different degrees of renal dysfunction in our study.

In our study, the histogram parameters based on DKI were also used to investigate their potential for assessing renal fibrosis. Except for skewness and entropy in cortex, the significantly negative correlations between the majority of *D* histogram parameters and interstitial fibrosis scores, as well as the significantly positive correlations between most of *K* histogram parameters and interstitial fibrosis scores were observed in CKD patients. Two studies, by Li et al. [31] and Liang et al. [18], also found a similar variation trend in rat model with unilateral ureteral obstruction and patients with IgA nephropathy. It revealed that the infiltration of cells, including typical fibroblasts and inflammatory cells, and collagen deposition result in restricted diffusion of water molecules in the interstitial space during fibrogenesis, which correspondingly reduces the values of *D* histogram parameters [32]. The progression of the glomerular lesion and tubulointerstitial injury and the gradual replacement of normal tissue by the expanding ECM and fibrotic tissue could make the microstructure more complex, which may aggravate the deviation of water molecule diffusion from the Gaussian distribution and correspondingly increase the values of *K* histogram parameters [33]. A higher *K* value typically indicates a more complex microstructure [27] and 90th percentile of *K* represents the region with the most intricate diffusion components. In the studies of Chen et al. [34] and Wang et al. [35], they have, respectively, found that the 90th percentile of *K* had the best diagnostic performance in predicting the grade of meningiomas and differentiating pathologic Gleason grade of prostate cancer among all histogram parameters. Similar with the above results, our study has demonstrated that the 90th percentile of *K* in cortex presents the strongest correlation with interstitial fibrosis scores and achieves the largest AUC for distinguishing the mild from moderate to severe fibrosis group. In addition, our study has shown that there are more significant histogram parameters in cortex than in medulla for assessing the different extents of interstitial fibrosis, which suggested that the progressive loss of normal structures with substitution by the ECM and fibrous tissue may have smaller effects on the medulla than the more vascularized cortex. Urinary proteins themselves may elicit proinflammatory and profibrotic effects and proteinuria is a strong marker for the progression of CKD, which was recommended to be used for regularly monitoring of CKD [36, 37]. In our study, the capability of 24 h-UPRO for evaluating renal fibrosis was investigated and our results have found that the *K*_90th_ of cortex exhibits better performance than 24 h-UPRO, and combining *K*_90th_ of cortex with clinical indicator 24 h-UPRO can further improve the diagnostic performance. Hence, the histogram parameter based on DKI combined with clinical indicator can get a preferable tool for comprehensive evaluation of renal fibrosis.

Entropy represents a statistical measure of irregularity of the voxel distribution, which could describe the heterogeneity of tissue from different perspectives [38]. Generally, higher entropy indicates more irregular distribution; lower entropy indicates more regular distribution. Our results have found that the entropy of *D* in cortex was significantly higher in the impaired eGFR group than that in the stable eGFR group and the entropy of *D* and *K* in cortex showed significantly negative correlations with eGFR. The results were in accordance with the investigation of previous research that entropy increased with the deterioration of kidney function [20]. Moreover, in the study of Fujimoto et al. [39], the researchers found that the entropy increased with an increase of the liver fibrosis stage, which was similar to our result that entropy of *D* in cortex showed a significantly positive correlation with the fibrosis scores. Skewness and kurtosis could, respectively, reflect the asymmetry and peakedness of signal intensity distribution in histogram. In a study on quantitative histogram analysis based on spatial labeling with multiple inversion pulses (SLEEK), Liang et al. [40] reported that there were significant differences in skewness and kurtosis between different renal function impairment groups. However, no significant differences in skewness and kurtosis between the two groups were observed in our study. This contradiction may attribute to the discrepancy between the selected sequence and grouping standard. Although there was no statistically difference between the stable and impaired eGFR group, the skewness of *D* in cortex was significantly negatively correlated with eGFR and showed significant positive correlation with fibrosis scores, which indicates that skewness has a certain value in evaluating renal function and fibrosis.

This study has several limitations. First, it is a study of a single institution with a small study population, further studies with a multi-center larger sample size are needed to confirm the results of this study. Second, this was a retrospective study with inherent biases in patient selection. Third, this study did not include normal volunteers for comparison with CKD patients. Fourth, this preliminary study used the first-order parameters of MR histogram merely, higher order parameters would be included in the future study.

In conclusion, this study demonstrates that histogram analysis based on DKI is feasible for evaluating the alterations of renal function and fibrosis in CKD patients. The *D*_mean_ of cortex had a good capability for differentiating renal dysfunction and the combination of *K*_90th_ in cortex and clinical indicator 24 h-UPRO achieved the best performance in the comprehensive evaluation of renal fibrosis. It could provide a noninvasive and accurate method of monitoring the progression of CKD and contribute to preventing CKD patients from poor prognosis.

## Abbreviations

AUC: Area under the curve
ADC: Apparent diffusion coefficient
BUN: Blood urea nitrogen
CKD: Chronic kidney disease
CKD-EPI: Chronic kidney disease epidemiology collaboration
CI: Confidence interval
D: Diffusivity
DWI: Diffusion-weighted imaging
DKI: Diffusion kurtosis imaging
eGFR: Estimated glomerular filtration rate
ECM: Extracellular matrix
FOV: Field of view
ICC: Intraclass correlation coefficient
K: Kurtosis
MRI: Magnetic resonance imaging
ROC: Receiver-operating characteristic
ROI: Region of interest
SCr: Serum creatinine
SS-EPI: Single-shot echo planar
SLEEK: Spatial labeling with multiple inversion pulses
T2WI: T2-weighted imaging
T1W: T1-weighted imaging:
TR: Repetition time
TE: Echo time
24 h-UPRO: 24 h urinary protein
